# *“What’s the point, when we’re already dead?”* Implementation challenges of COVID-19 public policies for indigenous peoples in the Peruvian Amazon: A sequential multi-method qualitative study

**DOI:** 10.1371/journal.pone.0340774

**Published:** 2026-01-09

**Authors:** Andrea Valdivia-Gago, Patricia J. García, Sherilee L. Harper, Angela Soria, Carol Zavaleta-Cortijo

**Affiliations:** 1 Intercultural Citizenship and Indigenous Health Unit (UCISI), Universidad Peruana Cayetano Heredia, Lima, Peru; 2 School of Public Health, Universidad Peruana Cayetano Heredia, Lima, Peru; 3 School of Public Health, University of Alberta, Edmonton, Canada; Norbert Wiener University, PERU

## Abstract

Peru issued multiple COVID-19 policies for the Amazon, yet how they worked in practice for Indigenous Peoples remains under-documented. We conducted a sequential multi-method qualitative study, reviewing 20 national and regional policy documents (Mar–Dec 2020) and interviewing 12 implementers, regional and local officials from the health sector (n = 8) and the Ministry of Culture (n = 4), plus one central-level culture representative, in Loreto and Junin. Triangulating top-down policy review with bottom-up practitioner accounts across two contrasting regions strengthened validity. Policies frequently lacked explicit intercultural guidance, clear monitoring indicators, and dedicated budgets. Implementers described budget misalignment, omission of specific health networks, delayed supplies, and connectivity barriers that fostered dissatisfaction and a perception that services prioritized data collection over care. Effects were most acute in remote and low-connectivity settings; Indigenous federations’ participation in Loreto sometimes mitigated challenges, while in Junin travel-fund constraints limited participation. Pandemic preparedness must institutionalize intercultural approaches and secure sustainable funding with clear accountability. Co-design with Indigenous organizations, ring-fenced implementation budgets, practical communication strategies, and routine monitoring are essential to protect Indigenous Peoples in future health emergencies.

## Introduction

The COVID-19 pandemic exposed and intensified long-standing health inequities, particularly for Indigenous Peoples in Latin America [1,2]. In Peru’s Amazon, geographic remoteness, language barriers, and limited primary care capacity intersected with an uneven policy rollout to shape who received protection and when. Against this backdrop, we examine how national COVID-19 policies aimed at Indigenous Peoples were implemented on the ground in the Peruvian Amazon during 2020.

Early studies from Peru and the broader region documented serious disruptions to routine care, locally led adaptations, and disproportionate COVID-19 burdens among Indigenous populations [3–7]. Comparative and regional analyses further highlighted structural drivers of inequity and called for responses grounded in intercultural approaches and the meaningful participation of Indigenous organizations [6,8]. In the Peruvian Amazon, community health workers and Indigenous federations often acted as first responders, though their efforts were only loosely coupled to national directives [7,9]. While this literature is rich on impacts and adaptations, systematic evidence remains limited on how policies intended to protect Indigenous Peoples were actually implemented during the first year of the pandemic, what was mandated, who was responsible, what resources were mobilized, and how measures translated into action across diverse Amazonian settings.

To analyse implementation, we combined a top-down and bottom-up approach [10]. From the top down, we reviewed national and regional policy documents to examine how regulations defined objectives, instruments, timelines, and responsibilities. From the bottom up, we drew on interviews with regional and local implementers to understand how these actors interpreted and adapted policies to their realities.

Our empirical focus is on Loreto and Junin, two regions that together capture key contrasts of the Peruvian Amazon. Loreto is largely riverine and sparsely connected by road [11]; Junin sits at the Andean–Amazon interface with mixed river and road access [12]. Both host diverse Indigenous Peoples and organizations yet differ in geography, population density, and health-system logistics [13]. Studying these regions enhances the representativeness of Amazonian diversity and enables cross-setting comparisons in policy implementation.

Before the pandemic, Peru already had some important, though unevenly applied, health policies in place. These included a national intercultural health policy with mediator programs, community health worker initiatives in Amazonian regions, and budgeting rules that determined how emergency funds were distributed to local areas and facilities. These existing structures shaped how the country responded to COVID-19. In some places, they made it easier to provide culturally appropriate outreach, while in others they slowed down fund distribution and last-mile delivery.

Our study uses a sequential multi-methods qualitative design to examine both policies documents and practices. A structured review of policy documents (March–December 2020) identifies all relevant norms and extracts information on target populations, intercultural elements, implementers, delivery mechanisms, and budgetary references. Semi-structured interviews with implementers in Loreto and Junin complement this by explaining how and under what conditions measures were or were not applied. Together, these methods reveal how policy aims translated into practice amid local constraints and adaptations.

Taken together, these elements guide our central question: how were COVID-19 policies for Indigenous Peoples implemented in the Peruvian Amazon during 2020, and what challenges and adaptations did implementers report in Loreto and Junin?

## Methods

### Study design

We use a sequential multi-method qualitative design to evaluate policies designed to serve Peruvian Indigenous Peoples. The desk review component focused on analysing national and regional policies focused on Indigenous communities, while the interview component concentrated on two regions of the country: Junin and Loreto. These regions were selected due to their largest number of Amazonian Indigenous populations, as identified by the 2017 National Peruvian Population Census [14]. Recognizing the importance of positionality in Indigenous Peoples’ health research, an Indigenous scholar (Dr. Zavaleta-Cortijo) co-led the analysis, contributing to both its design and interpretation.

### Data collection

#### Desk review component.

The Peruvian legal framework encompasses the Constitution, international treaties, laws, legislative and emergency decrees, and resolutions at various government levels, as well as local ordinances. To identify relevant legal documents, we used the governmental database of Peru (https://www.gob.pe/busquedas) using the term ‘*normas legales*’ and ‘COVID-19’ to identify the policy documents. In addition, we included all the documents from the collection of Regulatory Documents and Health Decrees in the context of COVID-19 (“*Documentos Normativos MINSA y Decretos de Salud en contexto* COVID-19”) accessed from the virtual library of the Ministry of Health of Peru (http://bvs.minsa.gob.pe/).

#### Eligibility criteria.

Eligibility criteria included: (1) documents released between March 15, 2020, the start date of the Public Health Emergency, to December 31, 2020; (2) to be implemented in the Amazon Peruvian region; (3) to include explicitly “Indigenous” or “ethnicity” in their content; and (4) the regulation’s objectives either reference the Indigenous Peoples or include considerations for them in the Loreto or Junin regions. We excluded documents that related to direct contracts, municipal ordinances or regulations for specific healthcare facilities, promotions awarded after death, cost and general expense estimates, approvals of donations, appointment of representatives, recognition and appreciation notices, and documents published with errors.

#### Implementer’s interviews.

Semi-structured interviews were conducted with regional and local officials and former officials involved in implementing the identified policies. A purposive snowball, non-probability strategy was used, suited to a small, highly specific population of COVID-19 policy implementers. Maximum variation was sought by region (Loreto, Junin), administrative level (regional, local, and one central-level actor), institution (regional health directorates, local health networks, Ministry of Culture), and role (management, logistics, community engagement).

Inclusion criteria were: (1) contracted public service during the study period and (2) direct participation in implementing one or more of the selected policies in the target regions/levels.

Given the specificity of the study population, policy implementers working on Indigenous-focused COVID-19 responses, this number provided both thematic saturation and diversity across institutions and roles. Initial participants (“seeds”) were contacted by phone or email through established collaborations of the Indigenous Health Adaptation to Climate Change program at Universidad Peruana Cayetano Heredia with Indigenous Peoples Health coordinators in Loreto and Junin, and via prior networks from Salud Sin Límites Perú. Interviewees could nominate additional eligible implementers. To mitigate homophily and network bias, multiple independent seeds were used per stratum, same-network referrals were limited, a sampling matrix was maintained, and proactive outreach targeted under-represented roles.

The interview guide was developed based on our theoretical framework (See S1 Appendix). During each interview, participants were presented with the policies identified in the desk review, tailored to their respective sectors, and asked to select one for discussion. The purpose of the interviews was to explore participants’ perceptions regarding the selected policies, including their success, degree of implementation, and factors influencing their outcomes. Specifically, the interview guide focused on: participants’ involvement in the policy’s implementation, the scope and feasibility of the policy, the distribution of responsibilities, ambiguities in the policy design, the level of consensus and commitment among stakeholders, the structure of implementation, and the incorporation of intercultural considerations. Participant recruitment began on 26 August 2021 and continued throughout September and October, concluding with the coordination of the final interview conducted on 21 October 2021. Interviews were conducted via Zoom, and all participants provided informed consent for audio recording.

### Data Analysis

#### Analysis of policy documents.

We analysed the identified policy documents using two main theoretical approaches: Top-down and Bottom-up [15]. Our analysis drew on Sabatier and Mazmanian’s Top-Down Implementation Theory and Lipsky’s Street-Level Bureaucracy model. The first emphasizes how central authorities shape policy outcomes through clear objectives, consistent procedures, and control over implementation. The second highlights the discretion of frontline implementers, those who adapt policies to local contexts and community realities. Together, these perspectives helped us explore how national intentions were interpreted and translated into practice within regional and local Indigenous settings during the COVID-19 response.

Building on these theoretical foundations, we used a framework that integrates top-down and bottom-up perspectives. Guiding questions were proposed to operationalise the theoretical approach [16]. Specifically, we asked: Who delivers the policy? What is the policy for? To the benefit of whom is the policy? Who is going to implement the policy? What resources are there to implement the policy? What is going to facilitate the implementation of the policy? These questions provided a structured approach to analyse the design of the public policies.

From the desk review, six policies referencing Indigenous Peoples and COVID-19 were identified for in-depth analysis. These were selected from the broader list of eligible documents and presented to interview participants according to their institutional sector to ensure familiarity and relevance. The selection reflected both systematic identification and contextual validation, acknowledging that policy visibility may have influenced inclusion.

#### Analysis of Implementer’s Interviews.

All the interviews were transcribed and analysed using ATLAS.ti software. A thematic analysis was conducted. Inductive codes were applied to meaningful text segments, and code reports were exported, summarized, and compared to identify recurring patterns. Related codes were grouped into broader themes capturing challenges and recommendations for culturally responsive policy implementation during pandemics [17,18].

### Ethical considerations

This study received ethical approval from the UPCH Institutional Review Board (IRB SIDISI: 205476). All participants provided written informed consent prior to their involvement in the study.

### Methodological considerations

Conducting interviews online may have constrained rapport and the depth of discussion compared to in-person interactions. Additionally, the snowball recruitment strategy may have introduced network bias despite our efforts to diversify participant sources. These factors were considered when interpreting findings.

## Results

**Fig 1:**
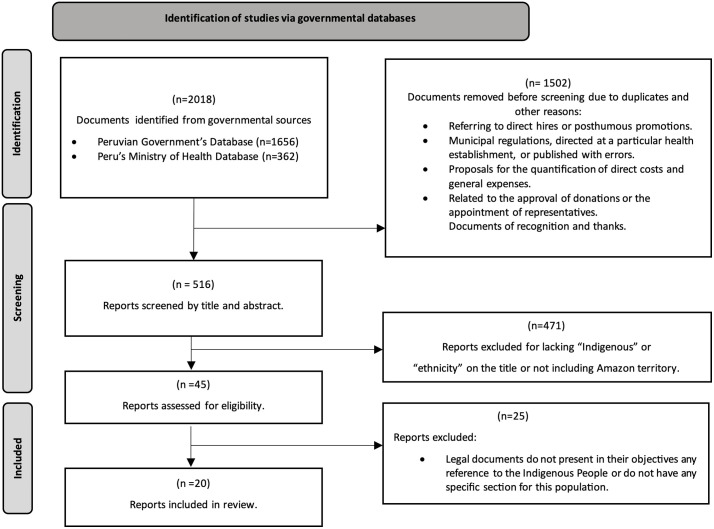
PRISMA flowchart of document selection process. A total of 2,018 documents were identified from governmental databases and screened for relevance. After removing 1,502 duplicates and unrelated records, 516 reports were reviewed by title and abstract. Of these, 45 were assessed for eligibility, and 20 met inclusion criteria for the final review.

We retrieved 2,018 documents from our initial database searches. After removing duplicates and non-policy documents (1,502), the documents were screened for the relevance of the title and abstract, resulting in the exclusion of 471 documents. We further evaluated 45 documents for full-text eligibility. Of these, 25 articles were excluded because they did not mention Indigenous People in their objectives or include a dedicated section for this population. Overall, 20 public health policies were included in this review. (See Fig 1).

**Fig 2:**
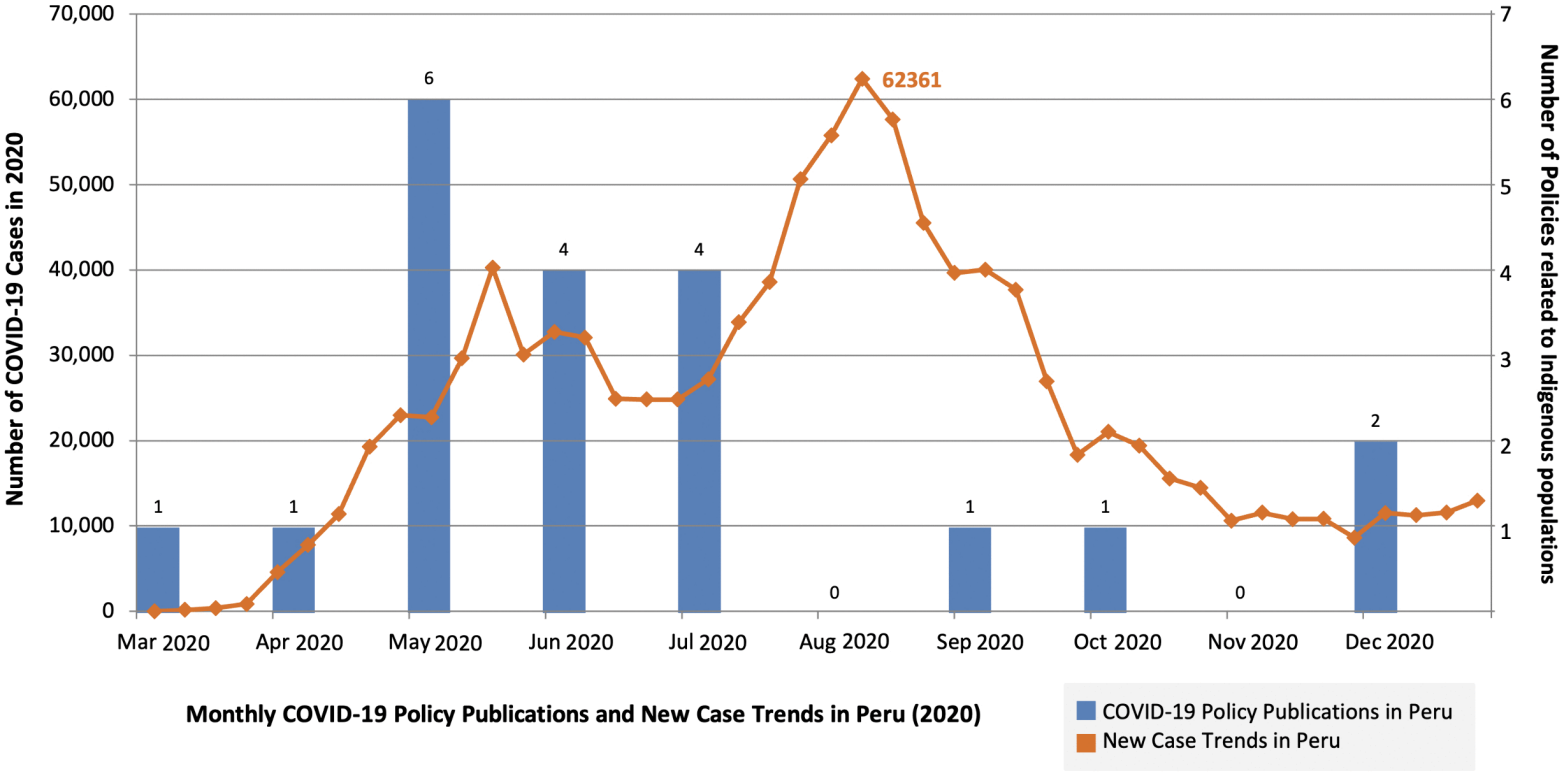
Monthly COVID-19 policy publications related to Indigenous populations and new case trends in Peru, 2020. Blue bars represent the number of COVID-19 policy documents addressing Indigenous populations (right y-axis), and the orange line shows new COVID-19 cases (left y-axis). Policy releases peaked in May (n = 6), preceding the highest case count in August (62,361). Policies from 2021−2022 were excluded, as they correspond to later pandemic phases beyond this analysis. *COVID-19 case data sourced from the Peruvian Open Data Portal: Datos Abiertos de COVID-19 (datosabiertos.gob.pe).*

Fig 2 illustrates the distribution of the 20 policies included in the review alongside the number of COVID-19 cases over time in 2020. The first policy emerged in March. An updated search of government databases on September 28, 2023, for the period 2021−2022 identified 116 new COVID-19 policies, only three [3] of which relate to Indigenous Peoples but were not considered in our analysis.

The Ministry of Culture led policies on Indigenous populations, followed by the Ministry of Health (MINSA, for its initials in Spanish). Most had a national scope, except for three (N° 308–2020-MINSA, N° 071–2020, N° 451–2020-MINSA), which were part of MINSA’s Intervention Plan in the Peruvian Amazon, targeting 10 regions with Indigenous and nearby rural populations.

Based on their objectives, we identified six major policy groups: (a) Initial responses during the first pandemic wave; (b) Health sector-led policies defining the Ministry of Health’s role; (c) Food assistance for Indigenous groups; (d) Policies for Indigenous groups in isolation; (e) Follow-up policies for COVID-19 measures; and (f) Culturally sensitive communication policies. Table 1 summarizes the policies by group.

**Table 1:** Policies related to the COVID-19 response for Indigenous communities in Peru, 2020 (N = 20). Summary of 20 governmental policies identified in the review, organized by issuing institution, date, and target group within Indigenous populations.

Group	Policy identified	Date	Institution
**Initial responses during the first pandemic wave**	Ministerial Resolution No.109-2020-MC	25-03-20	Ministry of Culture
	Legislative Decree No.1489	10-05-20	Executive Branch
	Supreme Decree No.004-2020-MC	29-05-20	Ministry of Culture
	Supreme Decree No.005-2020-MC	29-05-20	Ministry of Culture
**Health sector-led Policies**	Ministerial Resolution No.308-2020-MINSA	22-05-20	Ministry of Health
	Emergency Decree No. 071–2020	23-06-20	Executive Branch
	Emergency Decree 1020–2020	10-12-20	Executive Branch
	Ministerial Resolution No.212-2020-MINSA	21-04-20	Ministry of Health
	Ministerial Resolution No.451-2020-MINSA	01-07-20	Ministry of Health
	Ministerial Resolution No.314-2020-MINSA	23-05-20	Ministry of Health
	Ministerial Resolution No.22-2020-MINSA	21-07-20	Ministry of Health
**Food assistance for Indigenous groups**	Supreme Decree No.008-2020-MC	04-06-20	Ministry of Culture
**Policies for Indigenous groups in isolation**	Supreme Decree No.014-2020-MC	29-09-20	Ministry of Culture
	Ministerial Resolution No.0117-2020-MINAGRI	10-05-20	Ministry of Agriculture
	Ministerial Resolution No. 0152–2020-MINAGRI	28-06-20	Ministry of Agriculture
**Follow-up policies for COVID-19 measures**	Supreme Resolution No.005-2020-MC	17-06-20	Ministry of Culture
	Ministerial Resolution No. 386–2020-MINSA	15-06-20	Ministry of Health
**Culturally sensitive communication policies**	Ministerial Resolution No. 213–2020-DM/MC	30-08-20	Ministry of Culture
	Directorial Resolution No.169-2020-OGA/MC	02-12-20	Ministry of Culture
	Supreme Decree No. 010–2020-MC	30-06-20	Ministry of Culture

Policy information sourced from the official website of the Peruvian government (gob.pe/institucion/cultura/normas-legales).

### Policy documentary analysis

Policies show varying degrees of clarity in their provisions. While some provide specific guidelines for activities and budget management, others lack methodological tools for practical implementation. Challenges include unclear provisions regarding cultural and linguistic appropriateness of services for Indigenous populations, absence of criteria for defining policy intervention areas, and logistical oversights in implementing provisions, such as mask distribution and adapting facilities for specific contexts. The distribution of responsibilities showed lack of specificity.

Only half of the documents included the term “intercultural” in their content and a significant portion lack the consideration of the intercultural approach. Policies with an intercultural focus often include vague directives, like ‘adapt the provisions,’ without clear guidance. This is common in Ministry of Culture norms, which lack specific mechanisms for implementation.

Most policy documents analysed lacked monitoring mechanisms. One policy explicitly established them through biweekly meetings and monthly reports, while two others briefly mentioned monitoring guidelines. However, how regulations are communicated to lower levels remains unclear, as no specific indicators, goals, or additional resource allocations for implementation are mentioned. Additionally, a general lack of a dedicated budget for implementation is noted, with only three norms specifying funding sources.

### Implementers interviews

Out of the 14 individuals contacted for interviews, two did not respond, resulting in a total of 12 interviews. Six interviews were conducted in the Junin region, five in the Loreto region, and one with a representative from the Ministry of Culture at the central level. The participants included eight implementers from the Health sector and four from the Culture sector. The list of policies selected by participants for discussion is provided in S2 Appendix, which includes their full titles. For clarity and brevity, short titles are used to refer to these policies groups throughout the discussion section.

**Fig 3:**
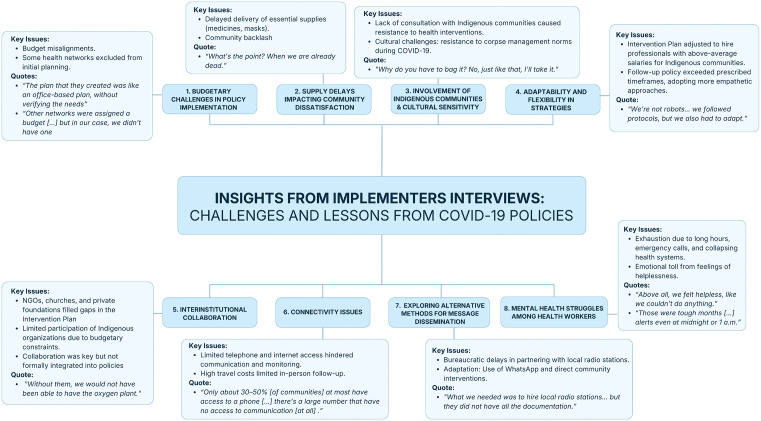
Key themes and insights from implementers’ interviews on COVID-19 policy implementation. *Figure developed by the authors based on interview data analyzed in the Results section.* The figure summarizes eight major themes that emerged from interviews with policy implementers, highlighting common challenges and lessons learned during the COVID-19 response in Peru. Themes include budgetary and supply issues, Indigenous involvement, flexibility in strategies, interinstitutional collaboration, communication barriers, alternative message dissemination, and mental health struggles among health workers.

From the interviews the following themes came out: budgetary challenges in policy implementation, supply delays affecting community satisfaction, Indigenous involvement and cultural sensitivity, adaptability and flexibility in strategies, interinstitutional collaboration, communication challenges, alternative message dissemination methods, and mental health struggles among health workers. For a visual representation of the key themes discussed, refer to Fig 3.

#### Theme 1: Budgetary challenges in policy implementation.

Interviewees described limited funding affecting implementation of the Intervention Plan and Isolation Centers policies. In the case of the Intervention Plan policy, interviewees reported challenges with fund distribution, for example:

*“The plan that they [the Ministry of Health] created was like an office-based plan, without verifying the needs, without checking what would actually be required to carry out the interventions*.” (Junin implementer).

Notably, many health establishments were initially omitted from budget planning, causing delays in their interventions. Implementers reported that their budgets were later included through policy modifications. Several interviewees stated that funding should be available from the outset. One interviewee noted:

“… *budget included funds to buy outboard motors and hire motorboat drivers [for areas without large rivers]. All of that had to be changed again. It was done in the end, but making those changes in the health system’s administrative platform, you know how long that takes.”* (Junin implementer)

Interviewees also reported budget constraints affecting the establishment and operation of isolation centres:

*“… we weren’t able to implement that [isolation centres] because we didn’t have a budget for it. Some other networks were assigned a budget to build their temporary isolation centres, but in our case, we didn’t have one.”* (Junin implementer)

#### Theme 2: Supply delays impacting community dissatisfaction.

Delays in obtaining essential supplies were common. Communities often received these items after prolonged waiting periods, leading to frustration and anger.

*“It wasn’t until the second week of October that the community was given soap, masks, and alcohol. This was met with resistance, people [in the community] said, ‘What’s the point now? You bring it when we’re already dead.’”* (Junin implementer)

#### Theme 3: Involvement of indigenous communities and cultural sensitivity.

Implementers described limited community consultation during policy design and instances of resistance (e.g., reluctance to testing or to isolation centres). For the Intervention Plan policy, references to intercultural approaches were reported as not always explicit, and some implementers relied on Indigenous community members for translation. One participant recalled national authorities describing the intercultural approach as secondary to containment. Informational videos from the Ministry of Culture were characterized as unclear:

*“We had to reach out … for translations. The Ministry of Culture was slow; messages weren’t clear, and only in Asháninka, despite many languages here.”* (Junin implementer)

Management of corpses was reported as a big cultural consideration.

*“Everyone [in the community] wanted to carry their deceased just like that, as if it was normal, ‘Why do you have to bag it? Why do you have to put a bag on it? No! I’ll just take it like that’ So we couldn’t change that*.*”* (Junin implementer).

In Loreto, under the COVID-19 Command policy, Indigenous federations monitored timelines/deliveries, convened regular coordination meetings, and acted as a first point of alerts from communities (e.g., requests for rapid brigades); pre-existing ties with federation leaders facilitated contact and case tracking. In Junin, participation was more limited, reportedly due to insufficient travel funds (see Theme 1).

This contrast shaped local coordination: in Junin, limited travel funding and less familiarity with the regional context reduced the ability of federation representatives to accompany brigades and relay information, affecting how implementation activities were organized on the ground.

#### Theme 4: Adaptability and flexibility in strategies.

Allocations for Indigenous-focused activities and hiring at above-average salaries were noted within the Intervention Plan policy. For the Follow-up policy, staff described practice that went beyond the call script:

*“… we’re not robots… we followed protocols, but we also had to adapt.”* (Loreto implementer)

Teams also reported extending follow-ups to two months (beyond the recommended four days) and using Administrative Services Contracts (CAS) for hiring.

#### Theme 5: Interinstitutional collaboration.

Although institutional collaboration across national and regional organizations was not recommended explicitly in the policies, implementers mentioned that they initiated collaboration practices when working with both the Intervention Plan and the COVID-19 Command policies. In the Intervention Plan policy, non-contemplated actors such as NGOs and private entities (such as churches and foundations) played a crucial role.

*“[The implementation of response actions] was 100% successful because we managed to achieve it with the support of the regional government and local governments. Without them, we wouldn’t have been able to get the oxygen plant or the oxygen tanks we needed at the time.”* (Junin implementer)

#### Theme 6: Connectivity issues.

Limited connectivity affected communication and monitoring. In the Follow-up policy, Ministry of Culture staff struggled to maintain contact with Indigenous communities in Loreto and Junin due to poor telephone and internet access. Travel costs were cited as a barrier to in-person visits.

“*It’s true that everything was handled virtually […] but out of the 400 communities we have in the central jungle, maybe only 30% or 50% at most, if we’re exaggerating, have access to a phone. There’s a large number of communities that have no access to communication*” (Junin implementer)

#### Theme 7: Exploring alternative methods for message dissemination.

Use of alternative communication methods was described. In the Advertising policy, coordination with multiple institutions was mentioned, along with delays linked to documentation requirements for local radio stations:

“*What we needed was to hire local radio stations, and many times we identified stations that weren’t exactly informal but didn’t have all the documentation the State normally requires.”* (Ministry of Culture - Central Implementer).

Despite challenges, implementers demonstrated flexibility by using alternative communication methods, such as WhatsApp and direct community interventions.

#### Theme 8: Mental health struggles among health workers.

Participants reported that long working hours, exhaustion, and the collapse of health services negatively affected both staff well-being and public health policy implementation. Interviewees described the strain of constant emergency calls about COVID-19 cases and deaths, as well as a deep sense of helplessness in the face of overwhelmed healthcare services.

*“Those were tough months because we didn’t have fixed schedules - we had alerts even at midnight or 1 a.m., reporting”* (Loreto implementer)

*“Above all, we felt helpless, like we couldn’t do anything […] the Health sector was overwhelmed [too].* (Loreto implementer)

## Discussion

The Peruvian government implemented a range of public health policies to prevent the spread of COVID-19 in Indigenous communities. However, many of these measures lacked clear guidance on intercultural and linguistic adaptation, delineation of responsibilities, and mechanisms for monitoring and funding. This ambiguity in roles and procedures suggests not only gaps in coordination, but also the absence of concrete implementation pathways capable of translating national intentions into effective local action.

Across regions, implementers repeatedly described resource constraints as the main barrier. Misaligned budget allocations and even outright omissions delayed execution, while supply lags fuelled community frustration and eroded trust. The success of many strategies depended on Indigenous involvement and cultural fit; however, intercultural guidance was unevenly put into practice, and national messaging was at times unclear. Where teams had room to manoeuvre, adaptability and flexible contracting helped them respond more quickly, but participation was uneven when travel funds were scarce. Cross-sector collaboration, including with non-state actors, often made the difference for securing critical inputs such as oxygen, yet connectivity gaps and high travel costs still hampered communication and monitoring in remote areas. Over time, sustained emergency workloads took a toll on staff well-being, with visible knock-on effects on implementation.

Beyond these persistent challenges, our findings underscore the gap between the documentary evidence-which reveals unclear implementation, fragmented monitoring, and insufficient budgeting and the firsthand experiences of implementers and Indigenous leaders, who described frustration, exhaustion, and a sense of abandonment. This integration of documentary and qualitative evidence offers a rare, multi-level perspective on how national directives translated unevenly into practice. While previous analyses have explored Indigenous health policy design in Latin America [4,19], this study provides the first in-depth examination of COVID-19 policy implementation for Indigenous Peoples in Peru, highlighting how structural barriers and intercultural gaps undermined an otherwise ambitious policy framework.

Our findings show that, although intercultural health principles were mentioned in official documents, their translation into practice depended heavily on local discretion, the availability of funds, and connectivity. The absence of consistent monitoring systems made it difficult to assess coverage, quality, and equity of interventions in real time. Interviewees repeatedly linked weak supervision and delayed budget transfers to feelings of uselessness. This convergence between policy review and field testimony illustrates how implementation failures are rooted not only in technical deficits, but also in historical patterns of exclusion and underinvestment in Indigenous territories [7,20].

International comparisons offer valuable lessons. New Zealand’s adaptation of COVID-19 communications for Māori populations and Canada’s community-level outbreak surveillance show that embedding Indigenous data governance and participatory monitoring can enhance accountability and trust [21,22]. For Peru and other Amazonian countries, developing a sustainable, intercultural monitoring system, co-designed with Indigenous federations, would institutionalize feedback loops and reduce dependence on ad hoc interventions. Similarly, ensuring that emergency funding lines are protected for Indigenous-focused activities would help maintain continuity beyond crisis cycles.

Sustainability requires moving beyond short-term pandemic response to institutional reforms that guarantee predictable financing, regular evaluation, and intercultural leadership within the health system [23,24]. Embedding intercultural indicators into national health budgets and public management instruments would not only enhance transparency but also reinforce the legitimacy of Indigenous participation in policy oversight. In this sense, sustainable pandemic preparedness must be seen as part of broader Indigenous self-determination in health governance.

Another key dimension emerging from our interviews was the mental health toll on frontline workers. Continuous exposure to emergency workloads, fear of infection, and inadequate institutional support led to high stress levels and burnout. These experiences point to an urgent need for occupational mental health programs tailored to intercultural and remote contexts, ensuring that both Indigenous and non-Indigenous health personnel can sustain their efforts during future crises [25–27].

### Limitations

This study has several limitations. First, the sample size was relatively small and focused on specific regions of the Peruvian Amazon, which may limit generalizability to other Indigenous contexts. Second, while the sequential multi-method qualitative design strengthened triangulation, the retrospective nature of the interviews may have introduced recall bias. Finally, the documentary review was limited to publicly available policies and regulations, which may not fully capture informal implementation practices or internal directives.

## Conclusion

Looking ahead, Peru can turn pandemic lessons into a durable framework for Indigenous health equity in the Amazon by addressing unclear directives, weak monitoring, unstable funding, and policies that ignore cultural and linguistic contexts that constrained implementers during COVID-19. Closing these gaps requires Indigenous leadership, co-governance with decision-making power, Indigenous-led oversight of preparedness and response, and data practices grounded in Indigenous data sovereignty. Policies should require practical tools: a co-designed training on intercultural approaches and rights for local health teams, multilingual communication protocols, and interoperable surveillance that publishes community-defined indicators. Financing should shift from ad-hoc grants to protected, multi-year lines supporting Indigenous organizations, community health workers, and locally managed supply chains.

Immediate steps policymakers can take include: (1) establish an Indigenous-led oversight mechanism with formal decision-making authority, created through binding agreements with regional health authorities; (2) roll out required, competency-based training on intercultural approaches and rights for local implementers within six months; (3) set up a rapid translation and interpretation service for Amazonian languages linked to all health alerts and guidelines; (4) publish a public dashboard with disaggregated, community-approved indicators and time-bound accountability milestones; and (5) earmark multi-year, protected budget lines for Indigenous organizations, community health workers, and locally managed supply chains.

## Supporting information

S1 Appendix Interview guide for officials or former officials at the regional and/or local level. (DOCX)

S2 Appendix Policies selected by implementers for discussion during the interviews. (DOCX)

S3 Appendix PLOS’ questionnaire on inclusivity in global research. (PDF)
