# Supplementary material for: “What’s the point, when we’re already dead?” Implementation challenges of COVID-19 public policies for indigenous peoples in the Peruvian Amazon: A sequential multi-method qualitative study
Source: PLoS One. 2026 Jan 9;21(1):e0340774. doi: 10.1371/journal.pone.0340774 (PMC12788662; doi:10.1371/journal.pone.0340774)
Supplement: S1 Appendix — (DOCX) [file pone.0340774.s001.docx]

**S1 Appendix. Interview guide for officials or former officials at the regional and/or local level**

| 1. **GENERAL INFORMATION** | |
| --- | --- |
| SEX |  |
| AGE |  |
| PROFESSION |  |
| INSTITUTION |  |
| TIME IN OFFICE |  |
| 1. **PARTICIPATION IN THE IMPLEMENTATION OF THE POLICY** | |
| *The interviewees will be read a list of the documents identified in Phase 1 that fall within their remit. Then they will be asked the following questions:*   - 1. Did you intervene in the implementation of any of the public policies that we have mentioned? (repeat the list if necessary ). If yes, which ones?, what did your participation consist of?   2. Have you intervened in the implementation of another that is not on the list and is address to the Amazonian Indigenous peoples public policy in the context of the COVID-19 pandemic? Which? What did your participation consist of?   3. From your perspective, do you consider that any representative or body of the Indigenous peoples should participate in the implementation of these policies that we have mentioned to you about COVID-19 and Indigenous Peoples? Because? | |
| *From now on, questions will be asked regarding the policy or policies in which the interviewees have indicated that they participated.* | |
| 1. **SCOPE AND VIABILITY OF THE POLICY** | |
| - 1. How ambitious and feasible do you consider the objectives of this policy to be?   2. Do they have sufficient resources (human and financial) to implement the actions proposed by the policy? If the answer is no, what do you need?   3. Are the timelines set for implementation appropriate? Because?   4. Do you consider that the activities proposed in the public policy will culminate in the expected results? Because? | |
| 1. **DISTRIBUTION OF RESPONSIBILITIES** | |
| - 1. Do you consider that the distribution of responsibilities is clear? How do your institution's responsibilities differ from those at the regional or national level (as appropriate)?   2. Is there overlapping of competences between the different levels of the sector? Because?   3. Are the activities that you must carry out in the implementation related to the competencies that your institution has as its mandate? Are there some activities that should not correspond to your institution? Which is it? | |
| 1. **POLICY AMBIGUITY** | |
| - 1. Do you consider that the objectives of this policy are clear, do they generate any doubts or confusion?   2. How much can the proposed activities be changed when implementing them?   3. What could be done to improve the clarity of the objectives of the policies that we have mentioned to you? | |
| 1. **CONSENSUS AND COMMITMENT AMONG STAKEHOLDERS** | |
| - 1. Who are the actors involved in the implementation of the policy? Find out if they are all public or if private parties are also involved.   2. How important is the implementation of this policy in relation to others that must be promoted in your sector?   3. Are all the actors in agreement and committed to the proposed activities and what each one should do? Because?   4. Do you think that there was a consensus between the implementers from the central offices and the field offices?   5. How committed do you feel that your office (your institution or your sector, as appropriate) was with the problem of COVID-19 and the Indigenous peoples of your region, when these regulations were issued?   6. In your opinion, what could be done to improve the consensus and commitment of the implementing actors with the policies that we have previously mentioned? (we read it to them again so they can remember it) | |
| 1. **IMPLEMENTATION STRUCTURE** | |
| - 1. Do they have manuals, documents or directives that direct the implementation? How clear are the implementation rules and procedures?   2. Is the performance of the corresponding activities subject to the actions or decisions of other actors? If the answer is yes, how?   3. Have they had the necessary resources for implementation at the necessary moment?   4. To which institution should you request resources and/or be accountable?   5. Do they have any other type of problem with funding or implementation resources?   6. How is the programmatic and financial monitoring of this policy exercised? Describe the system.   7. How much progress do you think has been made in the implementation of the policy? On a scale of 1 to 10, where 1 is "no progress" and 10 is "implementation is complete", how much progress would you say?   8. What have been the main barriers y retos (social, economic, political, geographical, etc.) that have influenced the implementation of the policy?   9. And what have been the main facilitating elements (social, economic, political, geographical, etc.) that have influenced the implementation of the policy? | |
| 1. **INTERCULTURALITY** | |
| - 1. Do you know the intercultural approach? Could you tell me what it consists of?   2. In your opinion, does this policy incorporate an intercultural approach? Because?   3. Have you implemented changes in the services to adapt them to the characteristics of the population?   4. Have you implemented dialogues with the population to make decisions about how the service should be?   5. Have any of the actions carried out implied the rescue of practices, techniques, instruments or traditional knowledge of the population?   6. From your perspective, what intercultural elements should be incorporated into policy documents to improve the implementation process? Can you explain to us please … | |
| 1. **PERCEPTION OF SUCCESS AND LEVEL OF IMPLEMENTATION** | |
| **About the policy [read the name of the policy]:** | |
| - 1. According to your perception: how successful has the policy “…” been considering that its proposed objective was “…”. Read and mark an option. - 100% successful in meeting your goal - 50% or more success in meeting your goal - Less than 50% success in meeting your goal - You have not been successful in meeting your goal   Because? and how? | |
| - 1. According to your perception: What level of implementation has the policy "..." to date. Read and mark an option. - 100% progress - 50% complete or more - Less than 50% complete - no progress   Because? and how? | |
