# Supplementary material for: “What’s the point, when we’re already dead?” Implementation challenges of COVID-19 public policies for indigenous peoples in the Peruvian Amazon: A sequential multi-method qualitative study
Source: PLoS One. 2026 Jan 9;21(1):e0340774. doi: 10.1371/journal.pone.0340774 (PMC12788662; doi:10.1371/journal.pone.0340774)
Supplement: S2 Appendix — (DOCX) [file pone.0340774.s002.docx]

**S2 Appendix. Policies chosen by implementers for the discussion during the interviews (each interviewee chose one, some were chosen by several interviewees)**

1. **Intervention Plan of the Ministry of Health for Indigenous Communities and Rural Populated Centres of the Peruvian Amazon**

(Short title: Intervention Plan Policy)

Ministerial Resolution No.308-2020-MINSA

1. **Guidelines for the Formation and Operation of the Indigenous COVID-19 Command at the regional level**

(Short title: COVID-19 Command policy)

Ministerial Resolution No.386-2020-MINSA

1. **Health Directive for Health Care in Temporary Isolation Centres and monitoring of COVID-19 cases in Peru**

(Short title: Isolation Centres Policy)

Ministerial Resolution No.314-2020-MINSA

1. **Health Directive to address the handling of corpses due to COVID-19 in localities with indigenous or native populations**

(Short title: Corpses policy)

Ministerial Resolution No.512-2020-MINSA

1. **Guidelines for the implementation of the alert strategy for the identification of suspected cases of COVID-19 in indigenous or native peoples […], and for the follow-up and monitoring during the medical treatment of the cases**

(Short title: Follow-up Policy)

Supreme Decree No.010-2020-MC

1. **Advertising Strategy Plan of the Ministry of Culture 2020**

(Short title: Advertising policy)

Ministerial Resolution No.213-2020-DM/MC
