# Supplementary material for: “What’s the point, when we’re already dead?” Implementation challenges of COVID-19 public policies for indigenous peoples in the Peruvian Amazon: A sequential multi-method qualitative study
Source: PLoS One. 2026 Jan 9;21(1):e0340774. doi: 10.1371/journal.pone.0340774 (PMC12788662; doi:10.1371/journal.pone.0340774)
Supplement: S3 Appendix — (PDF) [file pone.0340774.s003.pdf]

Inclusivity in global research

PLOS' policy on inclusivity in global research aims to improve transparency in the reporting of research performed outside of researchers' own country or community and ensures that PLOS publications reporting global research adhere to high standards for research ethics and authorship. Authors of relevant research articles may be asked to complete the questionnaire below, which outlines ethical, cultural, and scientific considerations specific to inclusivity in global research. This questionnaire may be requested when researchers have travelled to a different country to conduct research, if research uses samples collected in another country, research with Indigenous populations or their lands, or if research is on cultural artefacts. Researchers travelling to another country solely to use laboratory equipment will not normally be required to complete the questionnaire. However, the questionnaire can be requested at the journal's discretion for any submission – if you have been requested to complete this questionnaire by the PLOS journal you submitted to, please do so.

Please complete the questionnaire below and include this as a Supporting Information file with your manuscript. Note that if your paper is accepted for publication, this checklist will be published with your article in the supporting information files. Please ensure that you reference the checklist in the main body of your manuscript. We suggest adding a subsection 'Inclusivity in global research' to your Methods section and adding the following sentence: "Additional information regarding the ethical, cultural, and scientific considerations specific to inclusivity in global research is included in the Supporting Information (SX Checklist)"

The questions have been designed to be applicable to a wide range of study types, and there are subsections for both human subjects research and non-human subjects research. If any of the questions are not relevant to your research please mark them as "N/A" as appropriate.

Ethical considerations, permits and authorship

This section is applicable to all research types.

Provide details as to who granted permissions and/or consent for the study to take place in the Methods section of your manuscript. This should include the names of **all** ethics boards, governmental organizations, community leaders or other bodies that provided approval for the study. If individuals provided approval refer to these people by their role or title but do not list their name(s).

Reported on page number: 8

If there were any deviations from the study protocol after approval was obtained please provide details of these changes in the Methods section of your manuscript.

Reported on page number: N/A

Did this study involve local collaborators that are residents of the country where the research was conducted or members of the community studied? If you do not have any authors from said communities, please provide an explanation for this below.

Yes. This study was conducted in collaboration with local researchers based in Peru. Four of the five co-authors are affiliated with Peruvian institutions (Intercultural Citizenship and Indigenous Health Unit and School of Public Health at Universidad Peruana Cayetano Heredia in Lima, Peru), and an Indigenous scholar (Dr. Carol Zavaleta-Cortijo) co-led the design and analysis of the study, ensuring that perspectives from Indigenous Peoples' health research were integrated throughout.

Everyone listed as an author should meet PLOS' criteria for authorship and all individuals who meet these criteria should be included in the author byline, rather than the acknowledgements. For further information please see the journal's Authorship Policy.

Human subjects research (e.g. health research, medical research, cross-cultural psychology)

Did you obtain written informed consent from a representative of the local community or region before the research took place? How did you establish who speaks for the community? Details of written informed consent obtained from study participants should be reported separately in the Methods section of your manuscript.

Not applicable. Community-level written informed consent was not sought because this study did not involve direct data collection from Indigenous community members or the use of community-owned data. The research consisted of (a) analysis of publicly available national and regional policy documents and (b) semi-structured interviews with policy implementers (public servants) acting in their official professional roles in the health sector and the Ministry of Culture.

All interviewees provided individual written informed consent prior to participation, as described in the Methods/Ethics section of the manuscript. Because participants were recruited as institutional actors rather than as mandated representatives of specific communities, our institutional review board did not require separate community- or region-level authorization.

The study was co-designed and co-interpreted with an Indigenous health scholar based in Peru, whose longstanding work with Amazonian and Andean Indigenous Peoples helped ensure that the research questions and interpretation of findings were grounded in Indigenous health perspectives.

How did members of the local community provide input on the aims of the research investigation, its methodology, and its anticipated outcome(s)?

Given the policy- and implementer-focused nature of this study, local community members did not participate as a formal community advisory board or co-researchers specifically for this project. Instead, Indigenous and local perspectives informed the study indirectly through longstanding collaborations and the composition of the research team.

First, the study was co-designed and co-interpreted with an Indigenous health scholar based in Peru, who has extensive experience working with Amazonian and Andean Indigenous Peoples. This co-leadership helped shape the research questions, analytical framework, and interpretation of findings so that they reflected concerns previously raised by Indigenous communities about intercultural health and pandemic response.

Second, the recruitment of interview participants was built on existing collaborations of the Indigenous Health Adaptation to Climate Change program at Universidad Peruana Cayetano Heredia with Indigenous Peoples' health coordinators in Loreto and Junín, and on prior networks with Salud Sin Límites Perú. These partnerships, developed through earlier community-engaged work in the region, informed our decision to focus empirically on policy documents and policy implementers, in order to minimise additional research burden on communities already heavily affected by COVID-19.

When engaging with the local community, how did you ensure that the informed consent documents and other materials could be understood by local stakeholders?

Not applicable. This study did not involve direct data collection with Indigenous community members, and we therefore did not develop community-facing consent documents in Indigenous languages for this project.

Will the findings of the research be made available in an understandable format to stakeholders in the community where the study was conducted (e.g. via a presentation, summary report, copies of publications, etc.)? Please provide details of how this will be achieved.

Yes, this is planned. We plan to prepare a short, plain-language summary and policy brief in Spanish (2-3 pages) with the study aims, key findings, and practical recommendations, and share it with regional health directorates, local health networks, and Indigenous Peoples' health coordinators in Loreto and Junín. We will also circulate the final open-access publication, once available, together with a brief non-technical Spanish summary, to the Ministry of Health, the Ministry of Culture, and regional partners so that the results are available to them.

Non-human subjects research using specimens/ animals collected as part of the study, or those housed in archival collections. Examples include archaeology, paleontology, botany and zoology.

Did the permission you obtained from a local authority to perform the study include an agreement on access to outputs and benefit sharing? This may include procedures to enable fair distribution of the benefits and resources arising from the research performed. Please include any details of Prior Informed Consent and Benefit Sharing Agreements obtained. These may be required by field-specific regulations, for example the Convention on Biological Diversity (CBD) and the associated Nagoya Protocol.

N/A

If the material used in your study was imported, please A) provide the year it was imported and B) indicate whether permits were obtained to import/export the materials used, C) provide details of any permits obtained. If this information is not available, please indicate this.

N/A

If you used archival specimens, please state how the material used in your study was acquired by the institute it is held in and provide details of any permits obtained for the original excavations/ sample collection. If this information is not available, please indicate this.

N/A

How was the potential cultural significance of the materials collected in your study to local communities considered in your research design? Were Indigenous peoples and/or local researchers and institutions involved with archaeological excavations / collection of specimens? If so, please provide a description of their involvement.

N/A

If your manuscript includes photographs of human remains please indicate whether authors obtained permission from descendants or affiliated cultural communities to do so.

N/A
